# Importance of Mechanical Periodontal Therapy in Patients with Diabetes Type 2 and Periodontitis

**DOI:** 10.1155/2018/6924631

**Published:** 2018-09-25

**Authors:** Priscilla Naiff, Valéria Carneiro, Maria do Carmo Guimarães

**Affiliations:** ^1^Ph.D. Student, Faculty of Health Sciences, University of Brasilia, Distrito Federal, Brazil; ^2^Ph.D. Professor at Periodontics Division, University of Brasilia, Distrito Federal, Brazil

## Abstract

Periodontitis is an infectious and inflammatory disease of high prevalence worldwide and constitutes a significant oral health problem. It can lead to tooth loss. In addition, the local inflammatory process can cause the release of inflammatory mediators in the bloodstream and, consequently, contribute to the emergence of systemic effects as cardiovascular and diabetic complications. The purpose of this mini review is to alert health professionals about the risk that periodontitis represents for the onset or exacerbation of complications in individuals with type 2 diabetes mellitus and to emphasize that the mechanical treatment of periodontal disease and reestablishment of oral health are essential for the metabolic control of these patients. The periodontal therapy may help to reduce the risk of systemic complications in diabetes patients. Proper dental management should be suggested by health professionals, mainly from physicians to their patients, in order to improve the health conditions in these individuals.

## 1. Introduction

Diabetes mellitus (DM) is a metabolic disease in which the body does not produce insulin or cannot use it properly. There is an estimative that there are about 422 million people with DM worldwide [1].

The two main forms of the disease are type 1 (DM1) and type 2 (DM2) diabetes. Besides, other forms are also described in the literature, such as gestational diabetes, as well as other specific types of DM such as those associated with genetic disorders, among other factors. However, DM1 and DM2 affect most of the population, where 90% of the disease's cases are concentrated in type 2 [2].

In type 1 DM, beta-pancreatic cells are mistakenly attacked by the human's immune system. So, insufficient or no insulin is released. Consequently, glucose stays in the blood instead of being used as energy by the body [2].

Because most of the studies about the relation of DM and periodontitis are related to DM2 and this is the most prevalent type of diabetes, this review will approach the aspects only involved in the treatment of periodontitis in Diabetes mellitus type 2 patients.

DM2, a global public health problem, consists of a heterogeneous group of metabolic disorders that presents chronic hyperglycemia as a result of defects in the action or the insulin secretion. DM2 results from a combination of insulin resistance and inadequate compensatory response to insulin secretion, leading to a relative deficiency in the release of this hormone [3]. Insulin is the only hormone responsible for the reduction of blood glucose and is produced and released by the *β*-cells of the pancreatic islets of Langerhans [4].

The major complications of diabetes are microangiopathy, nephropathy, neuropathy, macrovascular disease, and delayed wound healing. Periodontitis is considered the sixth complication of DM [2].

Periodontitis is a primarily infectious and inflammatory disease caused by anaerobic bacteria (*Porphyromonas gingivalis, Treponema denticola, Prevotella intermedia, Prevotella nigrescens, Eikenella corrodens, Aggregatibacter actinomycetemcomitans*, among others) in association or not with other periodontopathogens, in dental biofilm. It affects teeth's protection and support tissues as gingiva and alveolar bone and can lead to dental mutilation [1, 5].

Periodontitis has been recently very strongly associated with the role of an overreactive immune system in the critical point of the periodontal disease development [6, 7]. Periodontopathogens stimulate, among other inflammatory mediators, the production of cytokines as interleukin 1 beta (IL1-*β*), interferon gamma (IFN-*γ*), tumor necrosis factor-alpha (TNF-*α*), and chemokines (CXCL-1, CXCL-8, CCL-5) by gingival epithelium, adhesion molecules, increased permeability of the gingival capillaries, and chemotaxis of neutrophils from the junctional epithelium to the gingival sulcus. This initial response, with the production of specific cytokines and chemokines, promotes the migration of an inflammatory infiltrate composed of perivascular T cells and monocytes to the connective tissue [6, 7]. If the cellular immune response fails to control the infection, recruitment of B cells that differentiate into plasma cells occurs. Plasma cells produce immunoglobulins (antibodies) that may confer protection to periodontal tissues, control the infectious process, or induce deleterious effects, leading to the destruction of connective tissue and promoting reabsorption of the alveolar bone. The effectiveness of this response varies between individuals and demonstrates importance in determining disease susceptibility [6].

Nowadays is well established the relationship between the progression of periodontitis and several factors, such as the presence of the periodontal pathogens, high levels of proinflammatory cytokines (IFN-*γ* and TNF-*α*), matrix metalloproteinases (MMP) and prostaglandin E_2_ (PGE_2_), and low levels of inflammation's inhibitory cytokines (IL-10), transforming growth factor beta (TGF-*β*), and tissue inhibitors of MMP (TIMP) [6, 8].

The susceptibility and extent of tissue destruction appear to be determined by the complex cytokine balance produced by the presence of numerous associations between periodontal microorganisms. When the host's response is exacerbated, it can lead to tissue damage, causing loss of periodontal support [6, 7].

Systemic diseases, such as diabetes, can also interfere with the periodontal condition, turning the prognosis of the associated diseases unfavorable [9]. Intravascular dissemination of microorganisms and their products throughout the body may occur because of the inflammation in the periodontal tissues. The total surface area of this periodontal inflammatory field is estimated to be the size of the palm of the hand. Immediate medical intervention should be done if one lesion of this size was on the skin. However, periodontitis is frequently ignored by health professionals, even though it may be associated with a range of systemic diseases and conditions [10].

The association of periodontitis with DM has been investigated and studies have shown that there is a definite correlation between both [11]. It can be observed that individuals with diabetes and inadequate glycemic control are more likely to develop severe periodontal disease and that periodontitis may interfere in the glycemic control of these individuals [12, 13].

## 2. Mechanisms of Action

Patients with uncontrolled diabetes can present with micro- or macrovascular complications. The prevalence of both complications occurs according to the type and duration of DM. Microvascular defects affect most intricately vascularized organs, as the retina (retinopathy), kidney (nephropathy), and peripheral nerves (neuropathy). The macrovascular defects affect large blood vessels, and consequently, some noble organs as the heart (cardiovascular disease), brain (cerebrovascular disease), and the peripheral arteries (peripheral vascular disease) [14]. Vascular disorders are usually progressive. The main problem in uncontrolled diabetes is the activation of the immune system that leads to micro- and macroangiopathies and other immune reactions contributing to all major organs failure. For example, nephropathy, begins insidiously, but over time, may contribute significantly to morbidity and mortality, resulting in severe damage to the organs. Cardiovascular diseases (CVDs) also account for increased morbidity and mortality in DM2 patients [15].

CVD usually occurs about two decades earlier among DM2 patients than in those without the disease [16, 17]. About 70% of individuals with DM2 die of CVD [18]. The combination of the duration of diabetes (>15 years) and prior CVD was associated with a 30-fold increased risk of fatal CVD [19].

Several factors can explain the mechanisms of periodontal destruction due to DM. Initially, the hyperglycemia state can directly favor the growth of periodontal pathogens, hinder or prejudice cellular functions and, consequently, host defenses. One pathogenic consequence of hyperglycemia in diabetes is an insufficiency in detoxification of reactive carbonyl compounds. Reactive carbonyl increases due to oxidative and nonoxidative reactions where carbohydrates and lipids lead proteins to chemical modification and then, at a late stage, to oxidative stress and tissue damage. This chronic and accelerated chemical modification of proteins is associated with the AGEs (advanced glycation end products) hypothesis which proposes that by increasing concentration of glucose in diabetes alters the structure and function of tissue proteins, contributing and precipitating the development of diabetic complications [20].

The immunological mechanisms mediating the effect of periodontitis on the control of diabetes have moderated level of support in the current literature. Most studies demonstrate that circulating proinflammatory mediators as TNF-*α*, CRP, and mediators of oxidative stress are elevated in patients with both diseases and these subjects tend to demonstrate higher dyslipidemia, reduced beta cell function, and elevated oxidative stress (that may act synergistically in worsening cardiovascular complications in diabetes) than patients with diabetes alone. Probably these mediators affect the control of DM, but there is no sufficient information from animal studies to support this possibility [21].

There is also a substantial increase in mediators as proinflammatory cytokines and secretion of collagen-degrading enzymes. Diabetes, through the formation of AGEs, can indirectly alter the union of the extracellular matrix, as well as cellular activities, amplifying inflammatory reactions, and decreasing cellular viability, which leads to deterioration of the healing process and potential change in periodontal tissues [22].

On the other hand, the mechanisms by which periodontitis promotes metabolic dysfunction are not yet fully understood. It is believed that in response to endotoxins such as lipopolysaccharide (LPS) produced by periodontal microorganisms, there is an augment in the production of proinflammatory cytokines, chemokines, reactive oxygen species (ROS), and C-reactive protein (CRP) that can alter lipid metabolism and insulin resistance, leading to hyperlipidemia and hyperglycemia [22]. Additionally, TNF-*α* has been identified as a potent insulin receptor blocker [23].

In severe untreated periodontitis, the ulcerated epithelium of the periodontal pockets has an estimated surface area of 8 to 20 cm^2^ [24]. This inflamed and ulcerated subgingival epithelial area of periodontal pockets constitutes a vast portal of entry for periodontopathogenic bacteria, their products, endotoxins such as LPS, and stimulated inflammatory mediators to reach the systemic circulation [25, 26].

Periodontal microorganisms as well as their antigens, when systemically dispersed, can cause significant systemic inflammation and contribute to DM complications. Leukocytes, endothelial cells, and hepatocytes respond to virulence factors with the secretion of proinflammatory mediators such as cytokines, chemokines, ROS, and CRP. If excessive, ROS release by phagocytes can reach circulation and cause systemic oxidative stress. CRP is a protein mainly produced by the liver as result of increased levels of TNF-*α* and IL-6 in the inflammatory process [27]. Cardiovascular disease has CRP as an independent predictor of its occurrence [28].

Data from a systematic review [29] concluded that human studies, animal experiments, and ex vivo cell culture studies provide evidence for elevated levels of interleukin-6 and interleukin-1*β* in periodontal tissues and crevicular fluid in patients with DM and periodontitis compared to systemically healthy patients.

Animal models with type 2 diabetes mellitus suggest that TNF-*α* plays an essential role in prolonging periodontal inflammation [29] and in the development of insulin resistance [23]. This mediator reduces the expression of glucose transporter type 4 (GLUT4) which is an insulin-regulated glucose transporter. TNF-*α* also induces serine phosphorylation of insulin receptor substrate-1 (IRS-1) that acts as an inhibitor of insulin receptor and down streams the signaling of phosphatidylinositol-3 kinase activation [23].

The increased release of proinflammatory cytokines (IL-1*β*, IL-6, and TNF-*α*), an altered RANKL/osteoprotegerin ratio, interactions between advanced glycation end products and their receptors, increased production of reactive oxygen species, and increased interaction between endothelial cells and leukocytes play a crucial role in the two-way relationship between diabetes and periodontitis. These complex changes, resulting from the presence of diabetes, modify the local inflammatory reaction in the periodontium, leading to a proinflammatory state in the gingival tissue and microcirculation [29]. With continued exposure, soluble antigens react with specific circulating antibodies to form immune complexes that amplify inflammation at the sites of deposition. Likewise, proinflammatory mediators, produced locally in the inflamed gingival tissues, can reach the systemic circulation. Proinflammatory cytokines in the circulation induce leukocytosis and acute phase proteins (e.g., CRP). In this context, the increase in the number of white blood cells is associated with an augmented risk of coronary heart disease, cardiovascular disease (CVD), atherosclerosis, thrombosis, and myocardial ischemia. This increase may be caused by the inflammatory nature of chronic infections such as periodontitis [30, 31].

Periodontitis may cause bacteremia and enhance atherosclerotic plaque formation because some microorganisms related to periodontal diseases were detected in atherosclerotic plaques [32, 33, 34]. However, other oral pathogens as *Streptococcus mutans* also have been found in atheromatous plaque samples [35]. Thus, it seems that the disruption of epithelial integrity from periodontal pockets may also provide a point of entry for nonperiodontal pathogens, as those usually found in caries-affected teeth.

Periodontal bacteria, as *P. gingivalis,* or their products can also interact with platelets (direct or *via* the vascular endothelium) and promote prothrombotic effects [36].

Proinflammatory cytokines, which have been reported to be associated with periodontitis, are also involved in atherothrombogenesis [37, 38]. Furthermore, periodontitis patients present many similar risk factors to those with CVD including age, lower socioeconomic status, and smoking [39]. This suggests that periodontitis and CVD may share common etiological pathways and that the association between both is plausible.

Periodontitis is a risk factor for atherosclerosis through endothelial activation. Bacterial products (LPS, outer membrane vesicles, or fimbriae), cytokines, and chemokines resulted from the infectious and inflammatory periodontal process fall into the bloodstream and may stimulate a superregulation of endothelial cell surface receptors in addition to the expression of adhesion on vascular endothelium. This promotes chemotaxis for circulating monocytes. These cells adhere to the activated endothelium. Due to molecular mimicry, immunoglobulins against specific bacterial proteins act as autoantibodies and induce apoptosis in the endothelium. The monocytes then migrate into the subendothelial space and differentiate into macrophages. There, they pick up oxidized low-density lipoprotein (LDL) and become foam cells. Apoptosis of LDL-loaded macrophages results in the accumulation of lipids in the subendothelial space, contributing to the formation of atheromatous plaques. In addition, invading periodontal pathogens induce the proliferation of smooth muscle cells in the formation of the intima and neointima. The extracellular matrix development and the extravasation of T lymphocytes result in the formation of a fibrous envelope covering the atheroma. The fibrous cap and its prothrombotic components are exposed after endothelial cell apoptosis. The enzymatic degradation of the extracellular matrix results in plaque rupture with consequent exposure of its prothrombotic components and formation of thrombi, leading to vessel occlusion. Clinically, this manifest as acute myocardial infarction, in the case of an occluded coronary artery, or a stroke in the case of an occluded cerebral vessel [40].

On the other hand, complications of DM2 because of periodontitis can be prevented or diminished if periodontal disease is treated. Studies have demonstrated that mechanical periodontal therapy can promote the reduction of inflammation's markers in the bloodstream (CRP, IL-6, among others) [41, 42, 43].

## 3. Importance of the Diagnosis of Periodontitis and Periodontal Debridement

Periodontal diseases have been associated with a reduced glycemic control in diabetes. Periodontitis increases the risk for the diabetes incidence in nondiabetic patients [21] as well as increases insulin resistance in patients with DM and disease complications, including mortality [44, 45].

The implications of periodontitis in the oral environment and maintenance of affected teeth, by themselves, would justify the relevance of seeking the complete understanding of its etiopathogenesis and, from this, implement active forms of individualized therapy. However, in addition to the implications of the disease in oral health, its meaning reaches systemic proportions, whose mechanisms are still not precise.

The effect of periodontitis on the control of DM type 2 has been studied, and there is indirect evidence to support biological mechanisms mediating this effect as reduced pancreatic islets *β*-cell function, elevated oxidative stress, and dyslipidemia. People with DM and periodontitis usually have elevated circulating proinflammatory mediators, like TNF-*α*, IL-6, CRP, and reactive oxygen species (ROS) that can interfere with diabetes metabolic control [44] and may act synergistically in worsening cardiovascular complications in diabetes [45].

Mechanical periodontal therapy involves the removal of bacterial agents from periodontium, supra, and subgingival calculus, by scaling and root planning with periodontal curettes or ultrasonic devices. It is the conventional treatment for periodontitis for resolution of inflammation from periodontal tissues and consequently, control of periodontal disease [46].

There is considerable evidence that nonsurgical periodontal treatment reduces oxidative stress, C-reactive protein level, and proinflammatory cytokines (i.e., tumor necrosis factor-alpha, interleukin-1*β*, and interleukin-6) [21, 42, 47, 48].

To monitor the success of the treatment of periodontal disease and the resolution of inflammation before and after therapy, besides radiographic examination, the oral clinical examination must include some essential periodontal indexes which are analyzed with the aid of a periodontal probe. The parameters usually analyzed are probing depth (PD), clinical attachment level (CAL), visible plaque index (PI), and gingival bleeding on probing (BOP) index [49].

In fact, if periodontitis truly has measurable effects on general health, treatment of this infection may alter the severity of the outcomes, with the resolution of inflammation.

The importance of periodontal treatment is not only to promote the reduction of local clinical inflammation, but it has also been associated with a subsequent decrease in serum levels of IL-6, TNF-*α*, CRP, and ROS [50–55]. This evidence supports the mechanistic link between periodontitis and diabetes through inflammatory mediators.

It is important to emphasize that diabetes can interfere with the homeostatic interaction between microorganisms and host at periodontal sites, where host immune response to diabetes can trigger a destructive inflammatory pathway against previously well-tolerated microorganisms. Experimental models demonstrate that the development of periodontitis in diabetic rats involves a high expression of proinflammatory cytokines (TNF-*α*, IL-1*β*, IL-6) and destructive tissue factors as advanced glycation end products (AGEs) without significant changes in commensal oral microbiota [56].

Patients with DM2 usually have glycated hemoglobin HbA1C elevation in serum, so current studies have shown that periodontal therapy can improve the control of HbA1C levels in patients with both diseases. Periodontal treatment can also successfully reduce circulating levels of TNF-*α*, CRP in patients with DM associated with periodontitis [57, 58]; however, research about the impact of successful long-term periodontal treatment does not exist and should be done. The magnitude of reported HbA1C reductions ranges from 0.27% to 0.48% at 3-4 months following periodontal therapy [58], which means the same quantity of short-term HbA1C reduction obtained to that often achieved by adding a second medication to a pharmacological regimen [59]. If such decreases can be sustained over the longer term after periodontal therapy, it may contribute to reduced morbidity and mortality associated with DM.

It is challenging to estimate the social cost of the morbidities related to patients living with diabetes. Many individuals are unable to continue their work activities because of chronic complications of the disease or remain with some limitations in their professional performance, causing significant losses regarding productivity. Thus, the control of periodontal disease in patients with DM through mechanical debridement (scaling and root planning) is crucial and may lead to better metabolic control and, consequently, to the improvement of the quality of life of these people.

It is of extreme relevance that health-care professionals, as physicians, to be aware of periodontitis and its implications for glycemic control and complications in individuals with diabetes. Diagnosis of periodontitis should be an integral part of a diabetes care visit. A periodontal examination should be done as part of their ongoing management of DM by a dentist. Even without diagnosed periodontitis initially, an annual periodontal review is recommended. All patients with DM should be provided with oral health education as part of their overall educational program [21].

In the other hand, dentists should pay attention in identifying both prediabetes and undiagnosed diabetes mellitus because periodontitis could increase the risk of many diabetes complications as retinopathy, nephropathy, neuropathic foot ulceration, cardiovascular diseases and mortality [60].

In conclusion, periodontitis and diabetes establish a two-way pathway, and each one, if untreated, could promote or exacerbate complications of each other. Periodontal screening must be part of the overall clinical examination of patients with diabetes and, if diagnosed, periodontal disease must be treated appropriately to avoid or exacerbate diabetes complications besides improving glycemic control in these individuals.

## Additional Points

*Clinical Significance.* To alert health professionals about the relation of diabetes mellitus and periodontitis and encourage them to conduct a multidisciplinary treatment/assistance.
